# Synthesis and optoelectronic properties of Cu_3_VSe_4_ nanocrystals

**DOI:** 10.1371/journal.pone.0232184

**Published:** 2020-05-05

**Authors:** Mimi Liu, Cheng-Yu Lai, Gurpreet Singh Selopal, Daniela R. Radu

**Affiliations:** 1 Department of Mechanical and Materials Engineering, Florida International University, College of Engineering and Computing, Miami, Florida, United States of America; 2 Institute National de la Recherche Scientifique, Centre Énergie, Matériaux et Télécommunications, Varennes, Québec, Canada; 3 Institute of Fundamental and Frontier Sciences, University of Electronic Science and Technology of China, Chengdu, PR China; 4 Department of Materials Science and Engineering, University of Delaware, Newark, Delaware, United States of America; University of Salento, ITALY

## Abstract

The ternary chalcogenide Cu_3_VSe_4_ (CVSe) with sulvanite structure has been theoretically predicted to be a promising candidate for photovoltaic applications due to its suitable bandgap for solar absorption and the relatively earth-abundant elements in its composition. To realize the absorber layer via an inexpensive route, printed thin-films could be fabricated from dispersions of nano-sized Cu_3_VSe_4_ precursors. Herein, cubic Cu_3_VSe_4_ nanocrystals were successfully synthesized via a hot-injection method. Similar with reported Cu_3_VS_4_ nanocrystals, Cu_3_VSe_4_ nanocrystals with cubic structure exhibit three absorption bands in the UV-Visible range indicative of a potential intermediate bandgap existence. A thin film fabricated by depositing the nanoparticles Cu_3_VSe_4_ on FTO coated glass substrate, exhibited a p-type behavior and a photocurrent of ~ 4 μA/cm^2^ when measured in an electrochemical cell setting. This first demonstration of photocurrent exhibited by a CVSe nanocrystals thin film signifies a promising potential in photovoltaic applications.

## Introduction

The class of sulvanites, with the formula Cu_3_MCh_4_ (M = V, Nb, Ta; Ch = S, Se, Te) is comprised of ternary copper chalcogenide semiconductors with the calculated optical band gap ranging from 1.19 eV to 2.60 eV, theoretically predicted p-type conductivity, and with potential in photovoltaic and optoelectronic applications. [1–3]

As suggested by current thin-film photovoltaic technologies, the absorbing layer of a thin-film solar cell should have an optical band gap of about 1.5 eV and a low hole effective mass. The sulvanites exhibit low hole effective mass of Cu_3_MCh_4_ decreasing along the chalcogen (Ch = S, Se, Te) series, and increasing with the transition metal group (V, Nb, Ta). [3]

Recently, the Kehoe group reported calculated band gaps of sulvanite structured Cu_3_MCh_4_. Specifically, the optical band gaps of Cu_3_VTe_4_, Cu_3_NbTe_4_, Cu_3_VSe_4_, Cu_3_TaTe_4_, and Cu_3_VS_4_ are 1.19 eV, 1.46 eV, 1.49 eV, 1.69 eV, and 1.72 eV, respectively, are suitable for photovoltaic applications. [3, 4]

While the telluride sulvanites Cu_3_MTe_4_ (M = V, Nb, Ta) present an appealing trend in regard to both their optical bandgap [3] and other applications, materials fabrication seems cumbersome due to the low reactivity and solubility of Te powder in the aliphatic solvents, [5, 6] hindering the wide application of Cu_3_MTe_4_ (M = V, Nb, Ta) materials. Thus, Cu_3_VSe_4_ and Cu_3_VS_4_ remain the sulvanite compounds that present most interest for photovoltaic applications. [7]

Solution-processed inorganic solar cells present a promising low-cost alternative to first-generation solar cells and could lead to technologies compatible with relevant terawatt capacities. [8]

The bottom-up approach for absorber fabrication utilizes nanocrystalline precursors that could be formulated in dispersion amenable to thin film deposition by printing methods. Recently, Chen at. al reported the first sulvanite synthesis in nanocrystalline form—Cu_3_VS_4_ (CVS) nanocrystals—obtained through a hot-injection synthesis. [9] Subsequently, Mantella et al. demonstrated the ability to control the size of nanocrystals, by synthesized cubic Cu_3_VS_4_ NCs with different sizes and narrow size distribution. The report also demonstrated the presence of an intermediate bandgap (IB) in Cu_3_VS_4_ through both theoretical calculations and experimental measurements. [10] Compared with photovoltaic devices with a single bandgap E_g_, semiconductors with an intermediate band can reduce the loss of incomplete absorption, since photons with energy below E_g_ can also be absorbed through transitions from the valence band (VB) to IB or from IB to the conduction band (CB). [11] In addition, because of the multiple-band transitions of VB-IB and IB-CB, the intermediate band semiconductors were proposed to have the possibility to reduce the thermalization effect, compared with single-band semiconductors. [12]

Therefore, it is predicted that semiconductors with intermediate bands have the potential to increase the conversion efficiency of a solar cell as the consequent effect of highly exceeding the Schockley-Queisser limit up to 63.1%. [10, 13] We anticipated that preparation of colloidal Cu_3_VSe_4_ could be achieved via similar synthetic methodologies.

In this work, we prepared Cu_3_VSe_4_ nanocrystals (CVSe NCs) by the hot-injection of Se precursor into cations precursor at 260°C. The obtained CVSe compounds exhibit the crystal structure of bulk CVSe as showed by X-ray diffraction and Raman spectroscopy. The nanocrystals show a cubic shape and particle size of 25 nm according to TEM and SEM analysis. The UV-Vis-NIR spectra of CVSe NCs show three distinct absorption bands that are similar to Cu_3_VS_4_ NCs. Additionally, the photocurrent of CVSe thin film on FTO substrates evidences its p-type semiconductor nature and great potential in the photovoltaic application.

## Materials and methods

### Materials

All chemicals used in the experiment were as received without further purification. Vanadium (IV) oxide acetylacetonate (VO(acac)_2_, ≥ 98%) was ordered from Merck KGaA. Selenium powder (Se, 99.99%) and oleylamine (OLA, 70%) were purchased from Aldrich. Copper(I) chloride (CuCl, 99.99%), trioctylphosphine oxide(TOPO, 90%), trioctylphosphine (TOP, 97%) and oleic acid (≥ 99%) were purchased from Sigma-Aldrich. Formamide (FA, 99%) and sodium sulfide (Na_2_S, anhydrous) was bought from Alfa Aesar. ACS grade chloroform (CHCl_3_, ≥99.8%), acetone (CH_3_OCH_3_, ≥99.5%), and methanol (CH_3_OH, 99.8%) were bought from Fisher Scientific, while ethanol (C_2_H_5_OH, 100%) was ordered from Decon laboratories. FTO Soda Lime glass substrates were purchased from MSE Supplies.

The crystal structure and purity of prepared CVSe NCs were performed by X-ray diffraction using Siemens Diffractometer D5000 (Cu Kα radiation, λ = 1.5405 Å) and Raman spectra conducted on the Raman Renishaw microscopy with 633 nm laser. JEOL 6330F Scanning Electron Microscope (SEM) combined with EDS was performed at 25.0 kV accelerating voltage to investigate the elemental distribution of the produced CVSe NCs. Philips CM200 Transmission electron microscopy (TEM) was used to determine the shape and size of the synthesized CVSe NCs. The chemical and electronic structure of the synthesized Cu_3_VSe_4_ NCs was determined by X-ray photoelectron spectroscopy (XPS) in a VG Escalab 220i-XL equipped with an Al Kα source. Photoluminescence (PL) spectra of CVSe NCs were conducted on PERKIN ELMER LS-55 Luminescence Spectrometer. The absorption spectrum of CVSe NCs was collected using Agilent Cary 5000 UV-vis-NIR spectrophotometer. The thermal stability of the Cu_3_VSe_4_ nanosheets was determined using a TA Instrument SDT-Q600 Simultaneous TGA/DSC.

### Preparation of CVSe NCs

In a typical synthesis, CuCl (1 mmol, 99 mg), vanadium (IV) oxide acetylacetonate (0.7 mmol, 185.5 mg), trioctylphosphine oxide (TOPO, 3 mmol, 1.31g), and 15 mL of oleylamine were loaded to a 100 mL two-neck round bottom flask, followed by degassing at 120°C for 30 minutes. Meanwhile, the Se source was prepared by dissolving 158 mg selenium powder (2 mmol) in a mixture of 5 mL oleylamine and 3 mL oleic acid and further evacuated at room temperature for 30 minutes. Upon degassing, both vessels were filled with argon. The Cu-V solution was heated to 260°C, and then the Se precursor was immediately injected at 260°C. Afterward, the reaction was maintained at 260°C for 1 hour. First, the product was transferred to the centrifuge tube with 4 mL TOP and ultrasonicated for 5 minutes to remove the residual Se. Then the precipitates were washed two times with a mixture of chloroform and ethanol at a volume ratio of 1:3. The precipitates were collected by centrifugation and dried overnight in a vacuum oven.

### Ligand exchange

The organic ligands attached to the surface of the synthesized colloidal nanocrystals are thought to block the charge transport when deposited in films and nanocrystal solids given that the organic ligands may generate insulating layers between the nanocrystals. [14–16]

In this work, a ligand exchange process was conducted to replace the organic ligands coordinated to CVSe NCs during synthesis (oleylamine, oleic acid, TOPO) with inorganic short ligands, S^2-^ by treatment with Na_2_S. [15] In a typical experiment, synthesized CVSe were suspended in toluene (8 mg/mL) and an amount of 10 mL of the CVSe suspension was added to a 10 mL of Na_2_S formamide solution (0.2 M). Next, the mixture was vigorously shaken for 1 minute and then hold until clear phases separate. The black CVSe NCs were transferred from the toluene phase to the formamide phase, indicating the successful ligand exchange. The clear toluene phase was removed, and a mixture of ethanol and nanopure water (v:v, 4:1) was added to precipitate the Cu_3_VSe_4_ NCs. The precipitates were washed twice with ethanol and nanopure water (v:v, 4:1), followed by purifying with a mixture of toluene and ethanol (v:v, 1:3). The resulting product was collected by centrifugation and dried in vacuum oven overnight for further use.

### Fabrication of CVSe thin film

CVSe dispersions (inks) were prepared by adding 25 mg of ligand exchanged CVSe NCs into 1mL ethanol and dispersed via ultrasonication for 10 minutes. 20 μL CVSe inks was deposited on FTO-coated glass by blade coating, followed by thermal treatment at 100°C for 1 minute in air, on a hot plate. The deposition process was repeated twice to fabricate the glass/FTO/CVSe thin film. Prior to deposition, the FTO substrates were cleaned in a sequence of distilled water, methanol, acetone, and ethanol through an ultrasonic bath for 10 minutes, respectively.

### Photoelectrochemical measurements

The photoelectrochemical response of CVSe thin film was tested by PINE reseach potentiostat connected with a three-electrode photoelectrochemical cell consisting of an Ag/AgCl reference electrode, a platinum counter electrode and a working electrode of CVSe-coated FTO substrate. All these three electrodes were held in the KCl aqueous solution (0.6 M) with a PH of 4.5. The current-voltage (J-V) curve of the CVSe thin film was swept from 0.6 V to 0 V in steps of 10 seconds light on-off with a sweep rate of 2 mVs^-1^ in the ambient atmosphere. The photocurrent of the CVSe thin film was produced by periodically irradiating LED light with 2000 lumens on the film for 10 seconds and shutting down the light for 10 seconds.

## Results and discussion

The CVSe NCs were characterized for crystallinity, purity, stability and composition.

**Fig 1:**
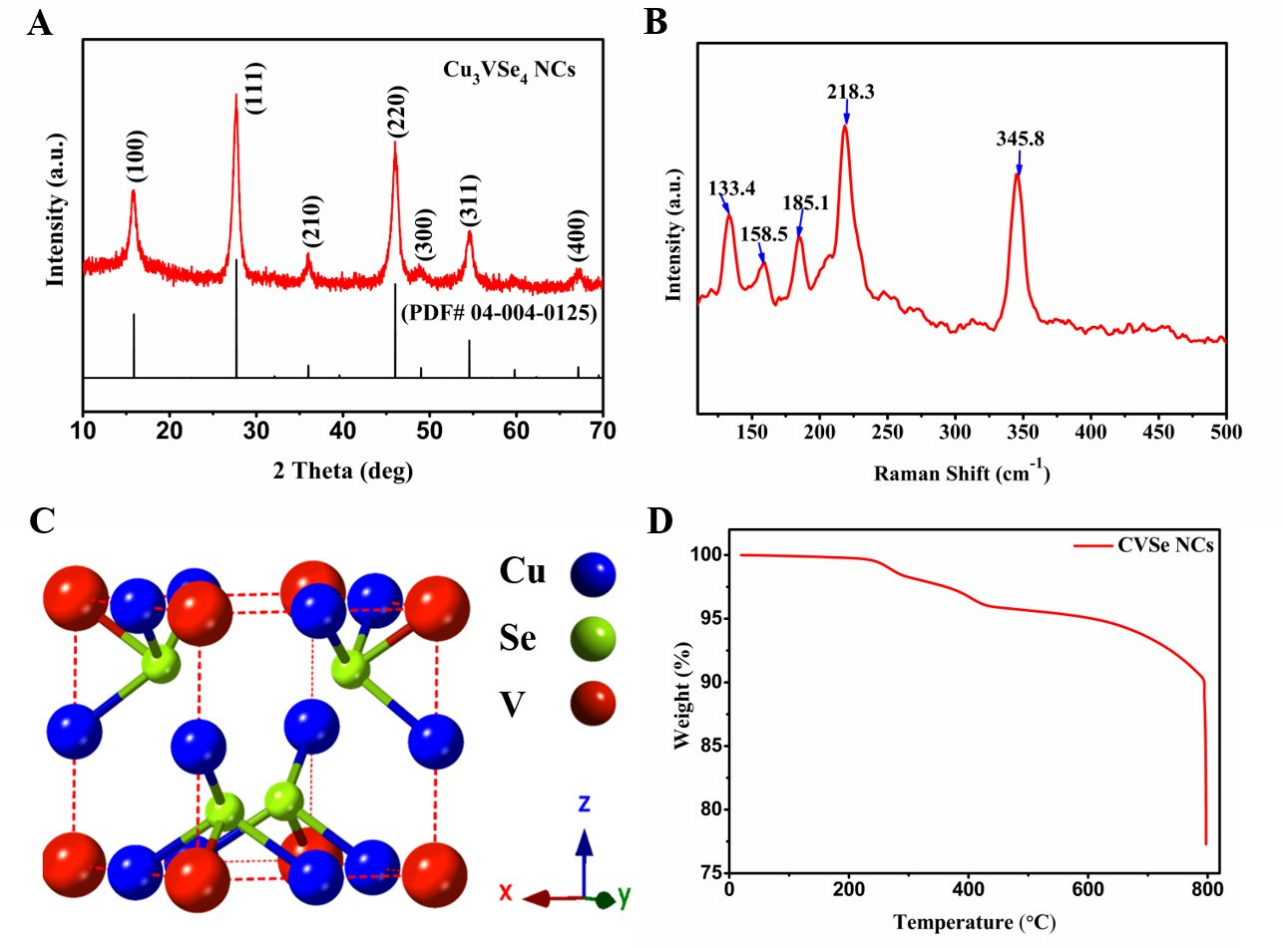
Characterization of CVSe NCs. (A) X-ray diffraction. (B) Raman spectrum. (C) Crystal structure. (D) TGA plot of synthesized CVSe NCs.

**Fig 1A** presents the X-ray diffraction of the synthesized CVSe, where each peak can be highly indexed to the cubic CVSe with a space group of *P* $\bar{4}3m$ (PDF# 40125). Raman spectrum of synthesized CVSe in **Fig 1B** exhibits five peaks at around 133.4 cm^-1^, 158.5 cm^-1^, 185.1 cm^-1^, 218.3 cm^-1^ and 345.8 cm^-1^, respectively. The crystal structure of synthesized CVSe is shown in **Fig 1C**.

The thermal stability of synthesized CVSe NCs was investigated using thermogravimetric analysis (TGA), where 18.5 mg of CVSe NCs were annealed in the range of 25–800°C at a ramping temperature of 20°C/min under argon atmosphere. The TGA curve in **Fig 1D,** conducted from 25°C to 800°C, shows two main mass loss events: the first weight loss, which begins at 250°C, could be associated with the evaporation of the residual organic ligand originated from the solution-phase synthesis, while the second mass loss, around 650°C correlates with the decomposition of CVSe NCs. To further understand the stability of CVSe under thermal stress, we annealed CVSe NCs at 650°C under argon atmosphere for 1 minute and 5 minutes, respectively. As shown in **S1 Fig**, the XRD pattern of CVSe NCs that was annealed for 1 minute exhibits pure CVSe phase, while the CVSe NCs annealing for 5 minutes displays a mixed phase of Cu_1.8_Se an CVSe, signifying that CVSe NCs start to decompose at 650°C.

**Fig 2:**
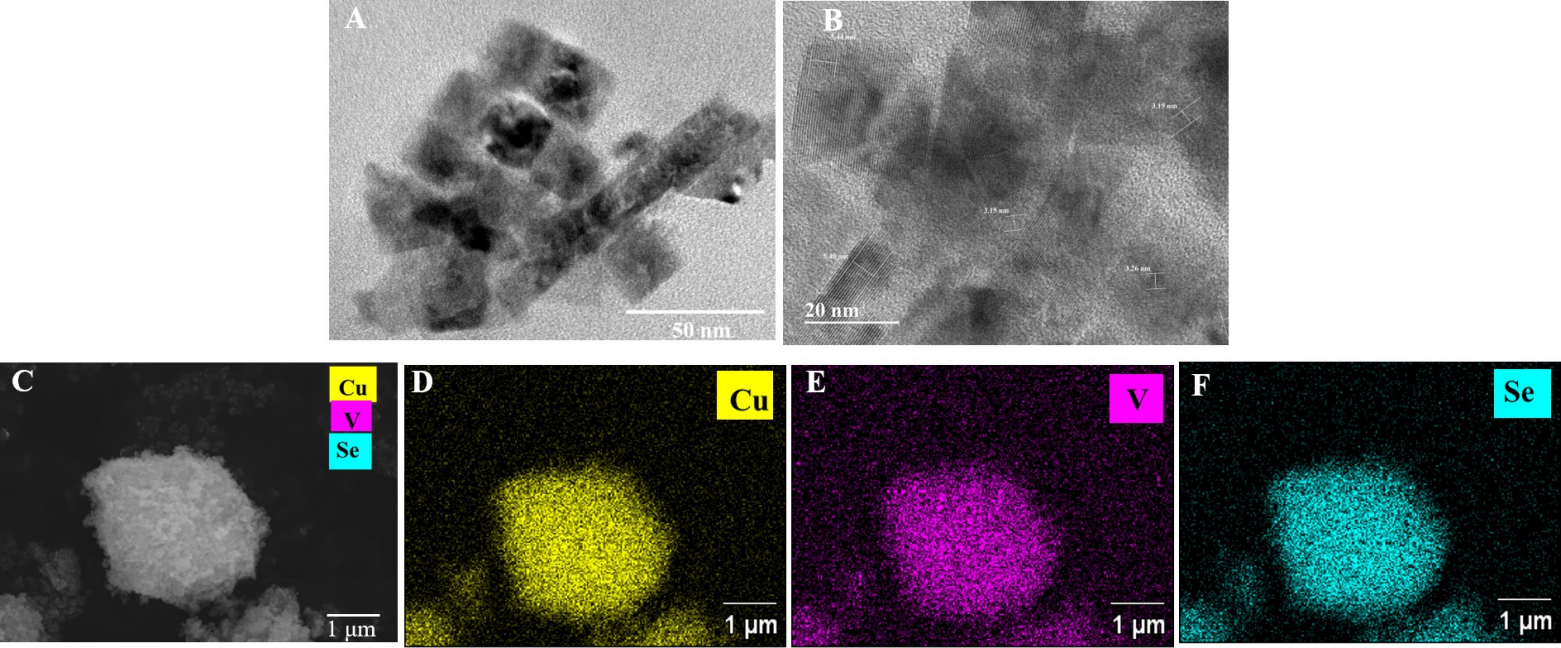
(A) Low-resolution TEM images. (B) HRTEM images. (C) SEM image of synthesized CVSe NCs. (D-F) SEM–EDS elemental mapping of CVSe NCs.

The low-resolution TEM image in **Fig 2A** reveals that the as-synthesized CVSe NCs have cubic shape. **Fig 2B** exhibits the high-resolution TEM (HRTEM) image, showing two measured interplanar spacing (d-spacing) of 0.32 nm and 0.54 nm, corresponding to the (111) plane and (100) of the cubic CVSe, respectively, illustrating the well-crystallized CVSe NCs. The elemental composition of the as-synthesized CVSe NCs was determined by SEM-EDS in **Fig 2C–2F**, showing a uniform distribution of Cu, V, and Se elements in the selected region. Consistent with XRD and SEM-EDS analysis, the purity of the obtained CVSe was also confirmed by Raman spectroscopy.

**Fig 3:**
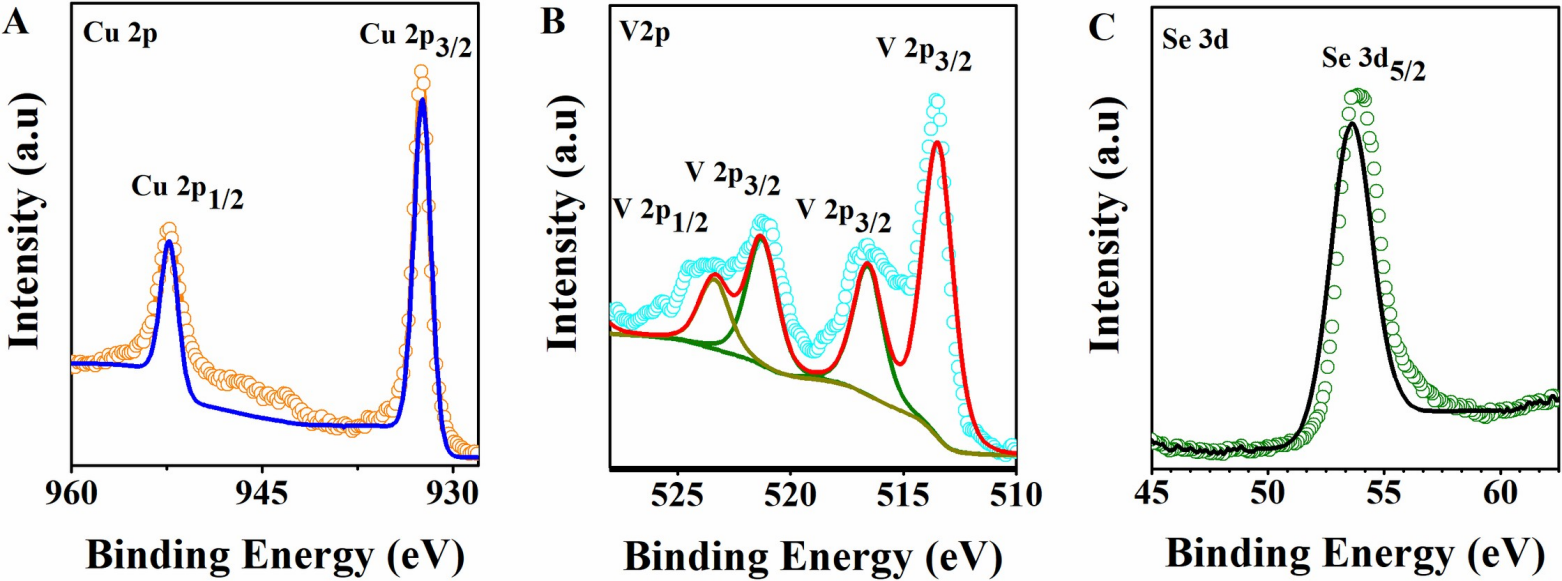
XPS spectra of the Cu 2p, V 2p and Se 3d peaks of CVSe NCs.

X-Ray Photoelectron Spectroscopy (XPS) was performed to determine the oxidation states of elements Cu, V, and Se present in the as-synthesized CVSe NCs. High-resolution XPS spectra of the Cu 2p, V 2p, and Se 3d orbitals are shown in **Fig 3**. The corresponding Cu 2p_3/2_ and Cu 2p_1/2_ core-level spectra of CVSe NCs in **Fig 3A** displays two peaks at 932.4 eV and 952.4 eV, respectively, suggesting that Cu is presented in Cu(I) state. [17–19] Base on the literature, the binding energies of V 2p appear at 516.7 eV(V 2p_3/2_) and 523.5 eV (V 2p_1/2_) with a separation of 6.8 eV in **Fig 3B** is close to V5^+^, [20, 21] whereas, the peaks at 513.5 eV and 521.3 eV with a separation of 7.8 eV corresponded to V 2p_3/2_ and V 2p_1/2_ in V 2^+^. [22–24] However, the XRD data shows only CVSe phase. In the study of Cu_3_VS_4_ material, Mantella et al. illustrated that the Cu_x_S nanoparticles and V-containing nanoparticles (V-NPs) were initially formed during the synthesis of the Cu_3_VS_4_ NCs. It is worth noting that the XRD of V-NPs showed amorphous status.[10] Thus, we speculated that some amorphous V-NPs could be formed in the synthesized CVSe NCs. The fitted spectrum of Se 3d in **Fig 3C** presents a peak at 53.8 eV which can be attributed to the Se 3d_3/2_ of Se^2-^. [25–27]

Overall, the XPS results demonstrate that the oxidation states of Cu, V, and Se in Cu_3_VSe_4_ NCs are the same as in bulk: +1, +5, and -2, respectively.

**Fig 4:**
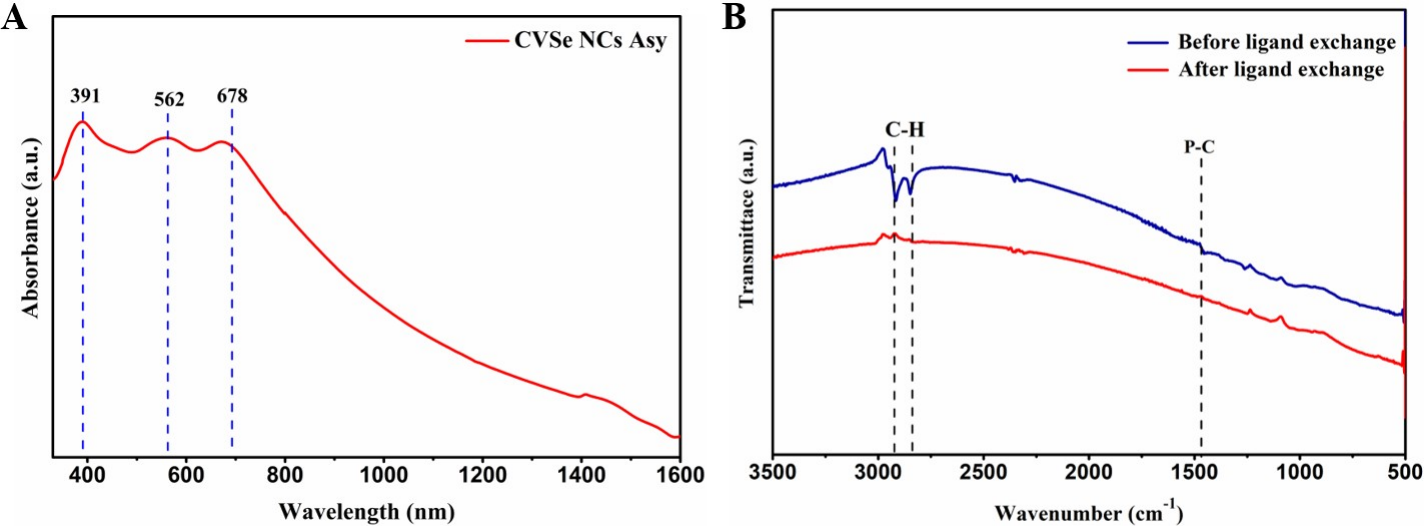
(A) UV-Vis-NIR spectra of the synthesized CVSe NCs in ethanol (B) FTIR spectra of the as-synthesized CVSe NCs (blue line) and CVSe with ligand exchange (red line).

**Fig 4A** shows the UV-Vis-NIR spectrum of synthesized CVSe NCs in ethanol, where three broad peaks were identified at around 391 nm, 562 nm, and 678 nm. When converting the wavelength to photo energy, these three absorption bands correspond to 3.17 eV, 2.20 eV and 1.83 eV which are in accordance with bandgap of VB-CB, VB-IB Ⅰ, and VB-IB Ⅱ, respectively. [28, 29]

Thin Film. Mitigation of residual carbon-based ligands on nanoparticles surface is reported as beneficial for thin film fabrication. Therefore, before depositing CVSe NCs inks on the FTO substrate, a ligand exchange process was conducted to remove the organic ligands, and their removal confirmed by infrared spectroscopy. Fig 4B displays the FTIR spectra of the as-synthesized CVSe NCs (blue line) and the NCs with ligand exchange (red line). The CVSe NCs were synthesized in the presence of oleylamine, oleic acid, and TOPO as solvent or surfactant. Thus, the characteristic band at around 1465 cm^−1^ corresponds to the P-C bond vibrational stretching; the characteristic doublet at around 2852 and 2925 cm^-1^ could be attributed to C-H stretching mode, suggesting that the synthesized CVSe NCs are capped with TOPO. These organic ligand-capped synthetic CVSe NCs underwent a ligand exchange with Na_2_S-formamide solution, and then the CVSe NCs transferred from chloroform to the formamide phase to generate inorganic ligand S^2—^terminated CVSe NCs, as shown in **Fig 4B** red line, where the C-H and C-P stretching apparently disappeared. The XRD pattern and Raman spectrum of the product after ligand exchange show pure CVSe phase, as shown in **S2 Fig** and **S3 Fig**.

**Fig 5:**
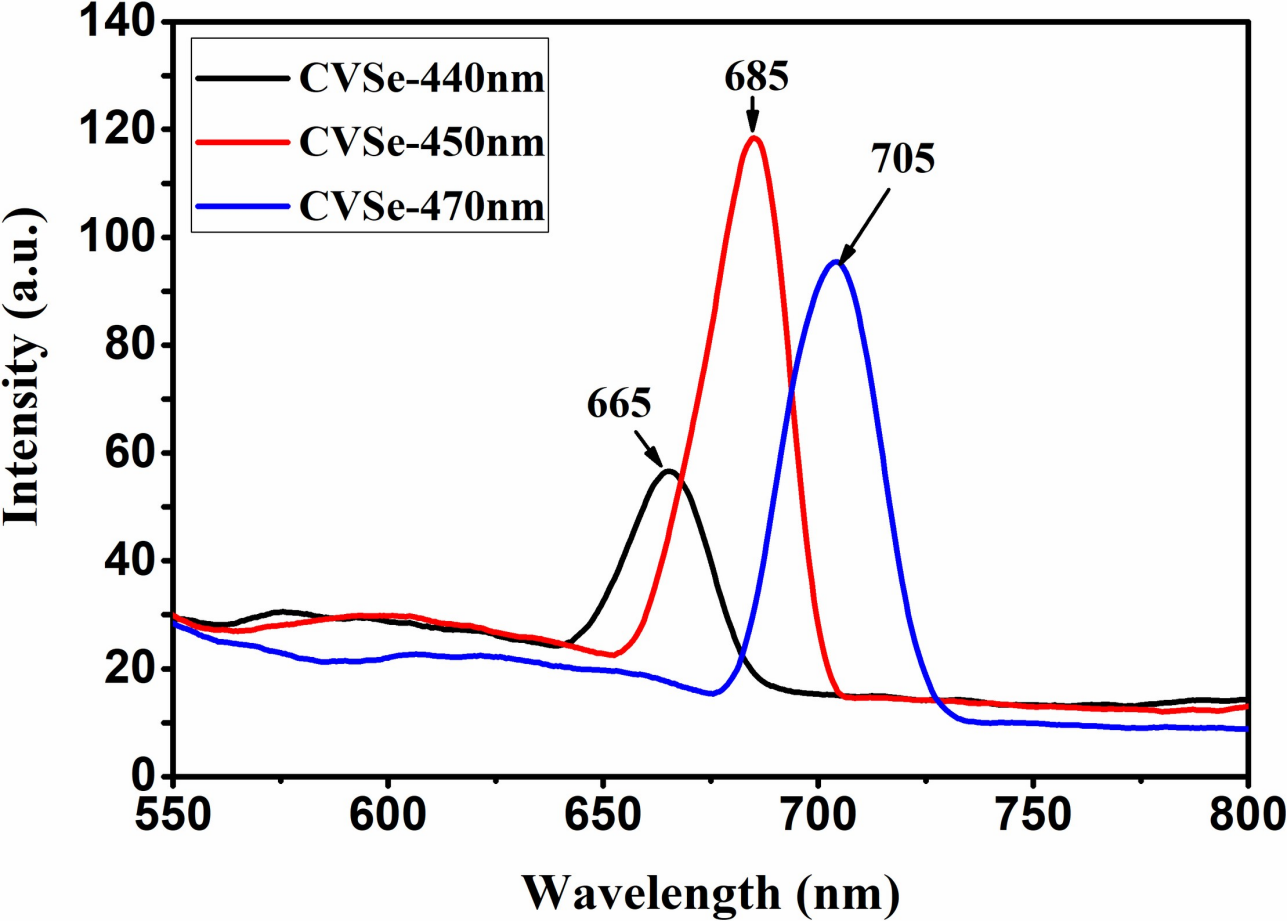
The photoluminescence (PL) spectra of synthesized CVSe NCs using different excitation wavelengths.

**Fig 5** shows the photoluminescence (PL) spectra of synthesized CVSe NCs using different excitation wavelengths. When the CVSe NCs are excited at the wavelength of 440 nm, the emission maximum locates at 665 nm. However, when increasing the excitation wavelength to 450 nm the emission maximum consequently shifts to 685 nm. As the peak of 685 nm possesses the highest intensity, thus, we prefer the 450 nm as the excitation wavelength of CVSe NCs. In other words, the optical bandgap of CVSe NCs is around 1.81 eV, in good agreement with the prediction from the above UV-Vis-NIR spectrum. Overall, the PL peak of CVSe NCs steadily shifts to the red region with the red-shift of the excitation wavelength. Possible explanation for this excitation-dependent PL phenomenon relates to the size distribution of CVSe NCs and the distribution of different emissive sites on the NCs. [30–33]

**Fig 6:**
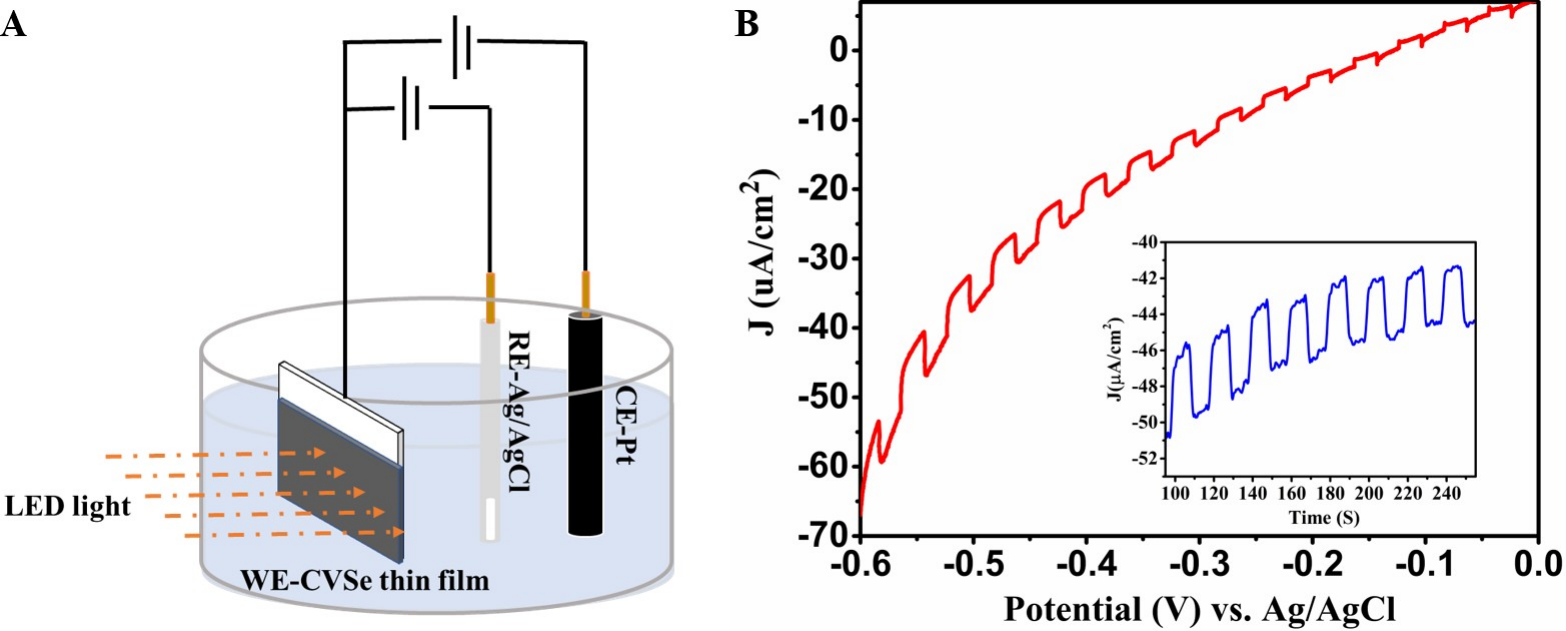
(A) Scheme of CVSe based three-electrodes photoelectrochemical cell. (B) Current-voltage (JV) curve of the CVSe thin film in KCl aqueous solution; insert graph is photocurrent response of the CVSe thin film in KCl aqueous solution at -550 mV.

To study the photoelectrochemical response of CVSe NCs we set up a three-electrode photoelectrochemical cell, as shown in **Fig 6A**. The current-voltage (J-V) curve of the fabricated CVSe thin film was investigated using a PINE research potentiostat by chopping the LED light with 10 seconds on and 10 seconds off, as shown in **Fig 6B**, where the current change progressively decreases along with the voltage bias towards positive, implying the p-type semiconductor characteristics of CVSe NCs. When the incident light illuminates the surface of the CVSe film, it generates electrons and holes. The photogenerated electrons transport to the interface of electrode/electrolyte and then reduce the H^+^ in the electrolyte to H_2_, whereas, the photogenerated holes transfer to the counter electrode through the external circuits and promotes the oxidation reaction (${2Cl}^{-}\to {Cl}_{2}\;or\;\frac{1}{2}H_{2}O\to H^{+}+O_{2}$). [34] In the chronoamperometry experiment, as shown in the inset diagram of **Fig 6B**, the photocurrent response of CVSe thin film in KCl aqueous solution was evaluated at -550 mV through several 10 seconds light on−off cycles where the CVSe thin film shows a photocurrent of ~ 4 μA/cm^2^ and high stability of the photocurrent. A potential reason for the low photocurrent of CVSe film could be the insufficient continuity of the CVSe thin film used. Both factors could affect the photoelectrochemical behavior of CVSe NCs; the film defects influences the transport of charge carriers. [35] Noteworthy, the CVSe film showed high stability in KCl aqueous solution, as it exhibits the correct Raman spectrum and active photocurrent response after being kept in the aqueous solution for one week. (**S4 Fig** and **S5 Fig)**.

To demonstrate the stable photoelectrochemical behavior of CVSe NCs, we measured the photocurrent response of three different CVSe thin films. All of the three different CVSe films display active photoelectrochemical behavior, as shown in **S6 Fig**. Thus, although the generated photocurrent is small, it is non-negligible and it is reproducible.

## Conclusions

We reported a facile solution-phase synthetic process to prepare the sulvanite-structured semiconductor Cu_3_VSe_4_ nanocrystals with cubic shape and an average particle size of about 25 nm. The UV-Vis-NIR spectra shows three main absorption bands in the UV-Vis range, indicating it possesses intermediate band. Thus, when compared to other solution-processed PV materials, CVSe NCs have an intermediate band, which is predicted to be able to absorb energies below the bandgap energy through two optical transitions from the valence to the intermediate band and from intermediate to the conduction band, resulting in enhanced conversion efficiency. Additionally, CVSe NCs possess relatively earth-abundant elements including copper, vanadium, and selenium. Interestingly, the PL peaks shift to red side when increasing the excitation wavelength, which could be attributed to a slight size distribution. The obtained Cu_3_VSe_4_ nanocrystals were subjected to a ligand exchange process with Na_2_S and then coated on FTO substrates to fabricate CVSe-FTO thin films. The CVSe thin films exhibited p-type photocurrents and photocurrents of ~ 4 μA/cm^2^ were recorded when immersing the film in a KCl aqueous electrolyte.

The photocurrent is stable and reproducible, suggesting that by optimizing both particles surface ligands, film fabrication techniques, and using an adequate device architecture, CVSe could become a significant player in solar photovoltaic applications.

## Supporting information

S1 Fig XRD pattern of CVSe NCs annealed for 1 minute (blue) and 5 minutes (purple). (TIF)

S2 Fig XRD pattern of as-synthesized CVSe NCs (bottom) and the CVSe NCs after ligand exchange (top). (TIF)

S3 Fig Raman spectra of as-synthesized CVSe NCs (bottom) and the CVSe NCs after ligand exchange (top). (TIF)

S4 Fig Raman spectra of the CVSe thin film. Dark line is the Raman spectrum of fresh CVSe thin film; Red graph is the Raman spectrum of the CVSe film that was kept in KCl aqueous solution for 1 week.
(TIF)

S5 Fig Current-voltage (JV) curve of the CVSe thin film in KCl aqueous solution. Dark line is the JV curve of fresh CVSe thin film; Red line is the JV curve of CVSe film kept in KCl aqueous solution for 1 week.
(TIF)

S6 Fig Photocurrent response of three different CVSe thin films. (TIF)
