# Supplementary figures and images for: Synthesis and optoelectronic properties of Cu3VSe4 nanocrystals
Source: PLoS One. 2020 May 5;15(5):e0232184. doi: 10.1371/journal.pone.0232184 (PMC7199925; doi:10.1371/journal.pone.0232184)

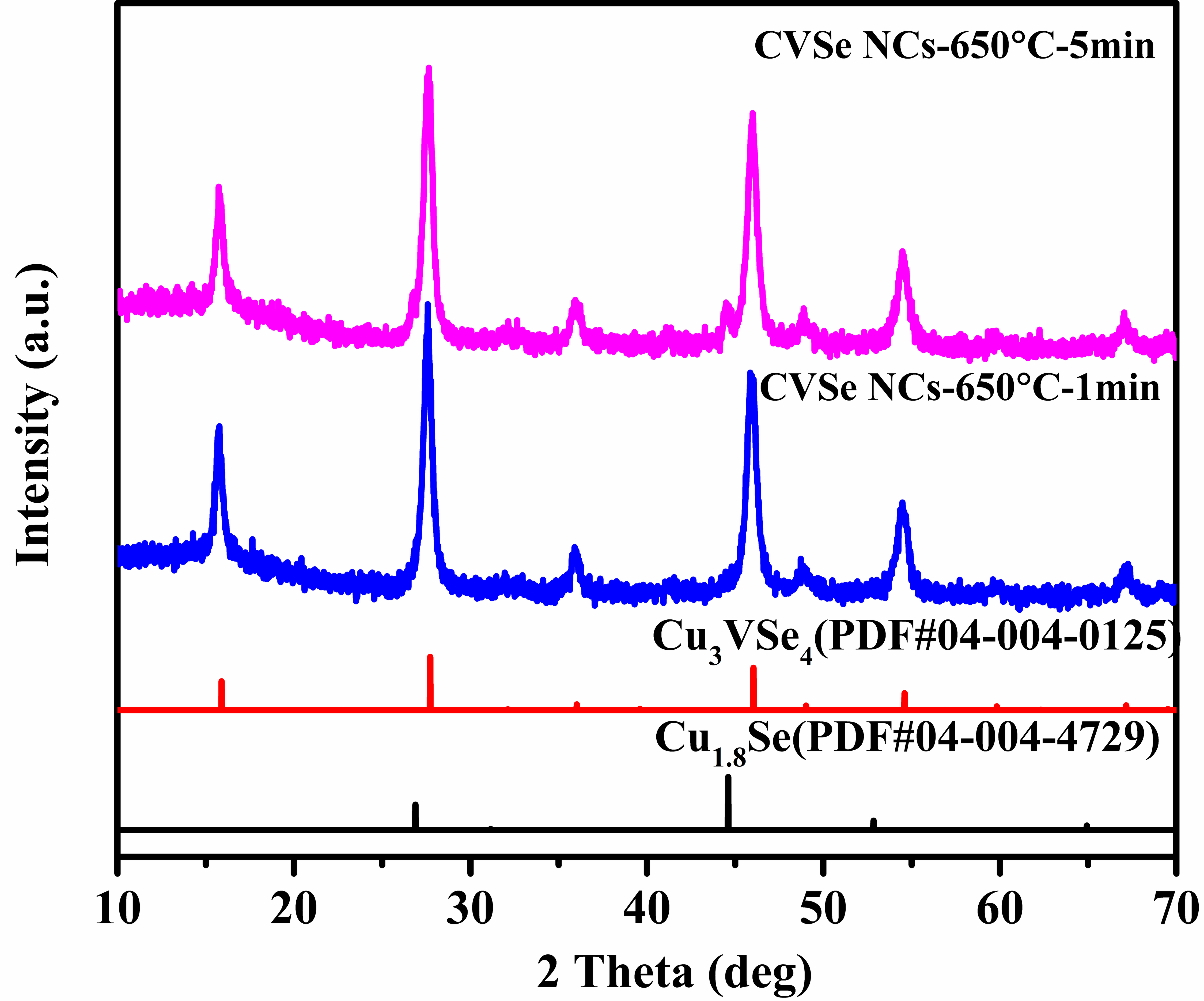

Supplement: S1 Fig — (TIF) [file pone.0232184.s001.tif]

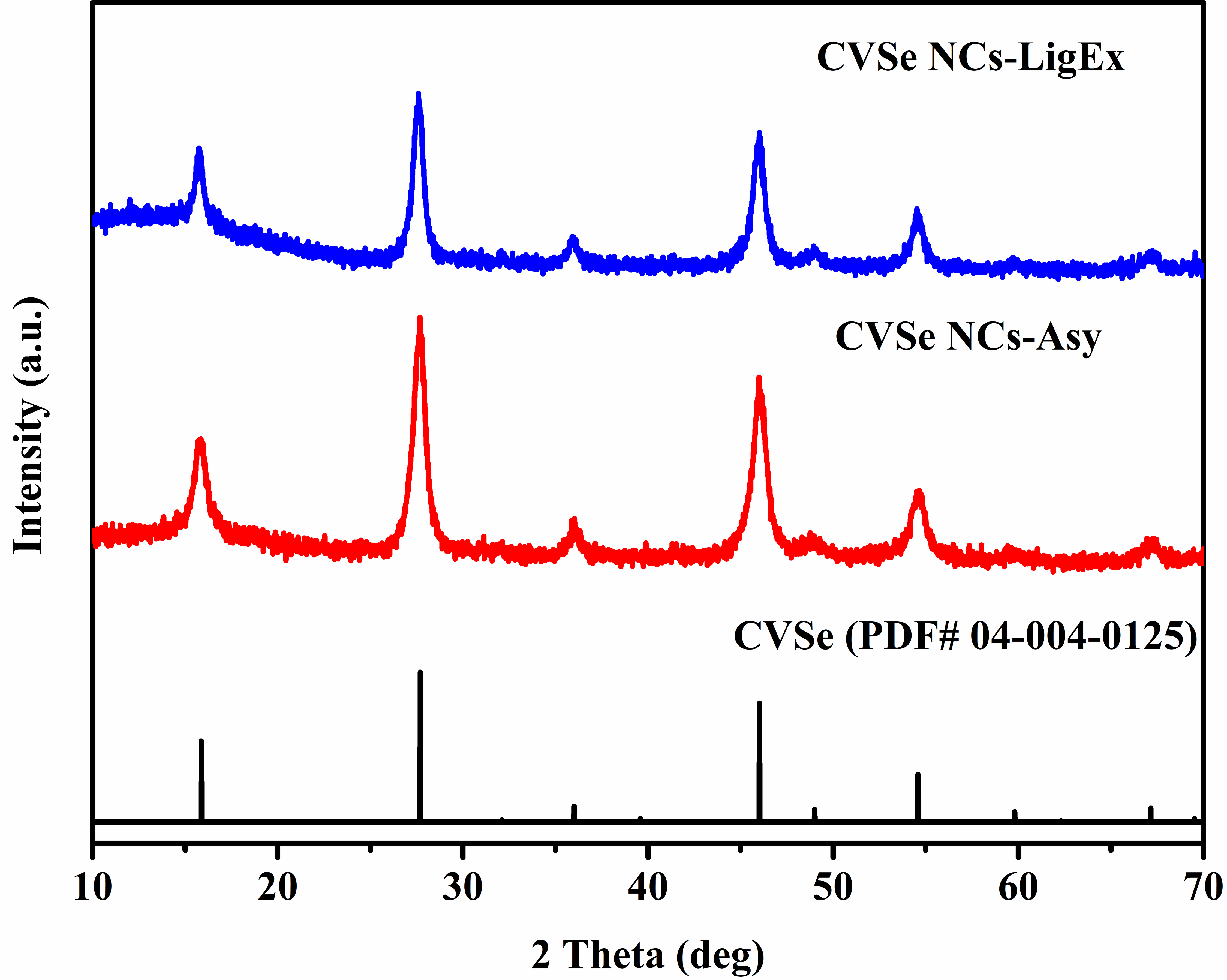

Supplement: S2 Fig — (TIF) [file pone.0232184.s002.tif]

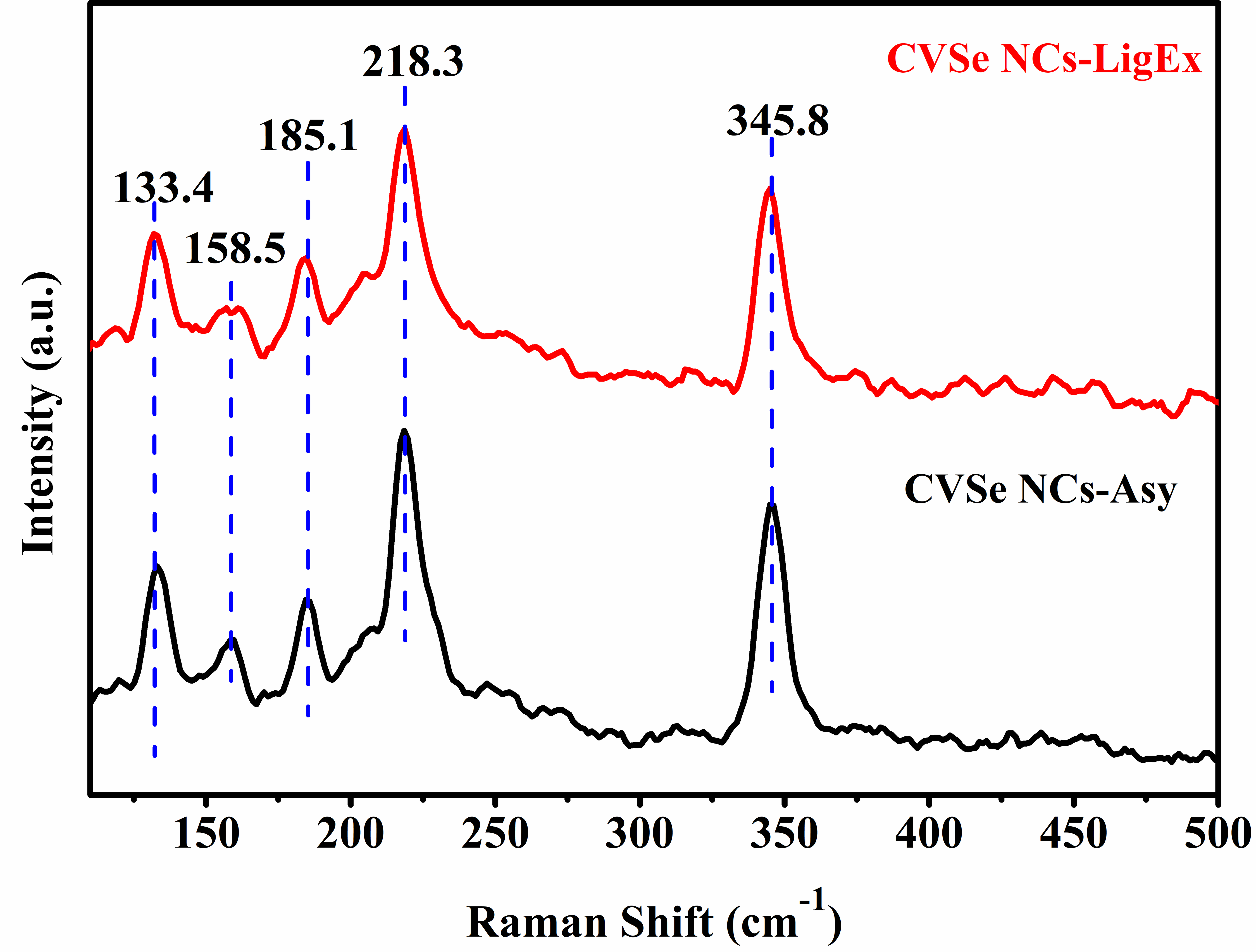

Supplement: S3 Fig — (TIF) [file pone.0232184.s003.tif]

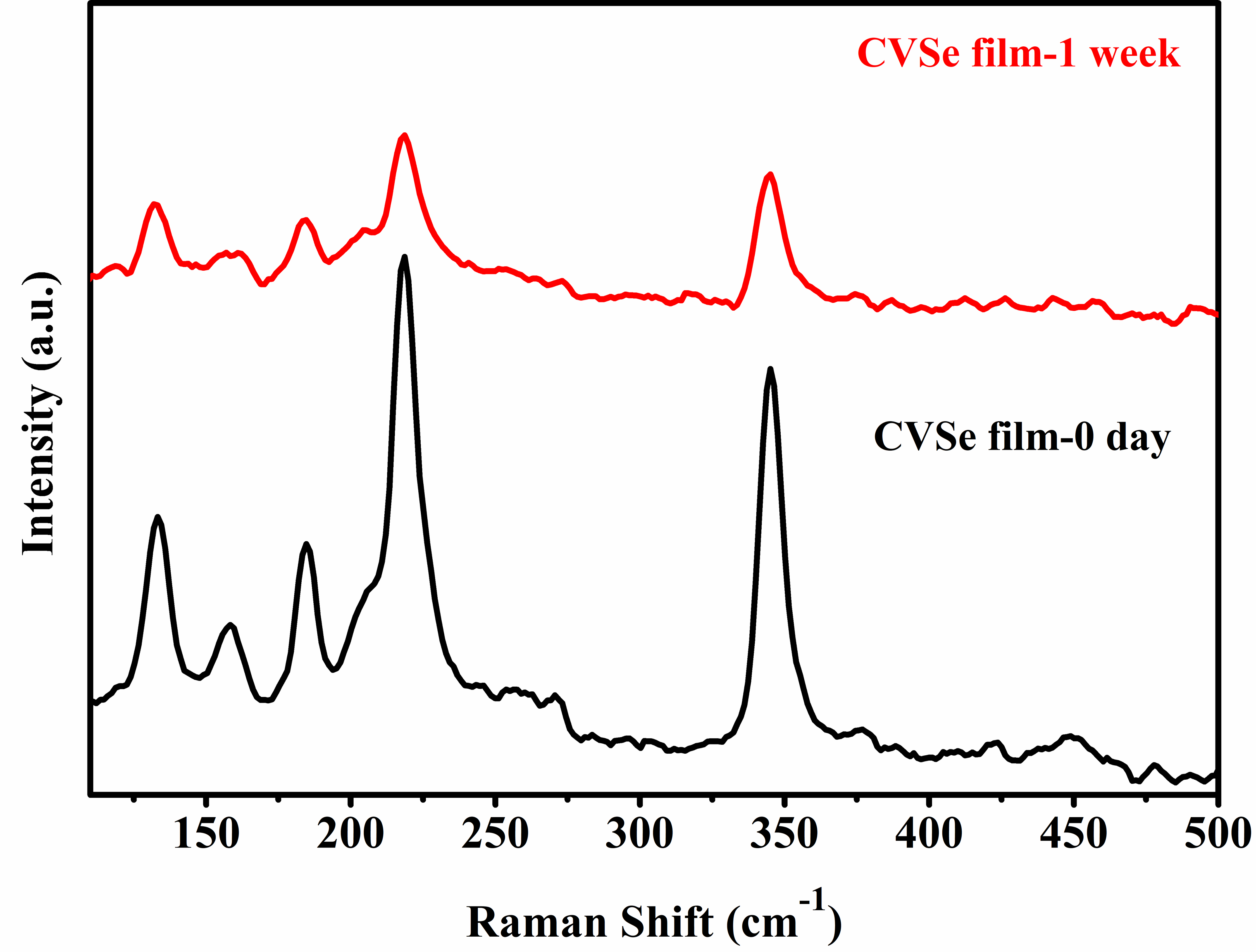

Supplement: S4 Fig — Dark line is the Raman spectrum of fresh CVSe thin film; Red graph is the Raman spectrum of the CVSe film that was kept in KCl aqueous solution for 1 week. (TIF) [file pone.0232184.s004.tif]

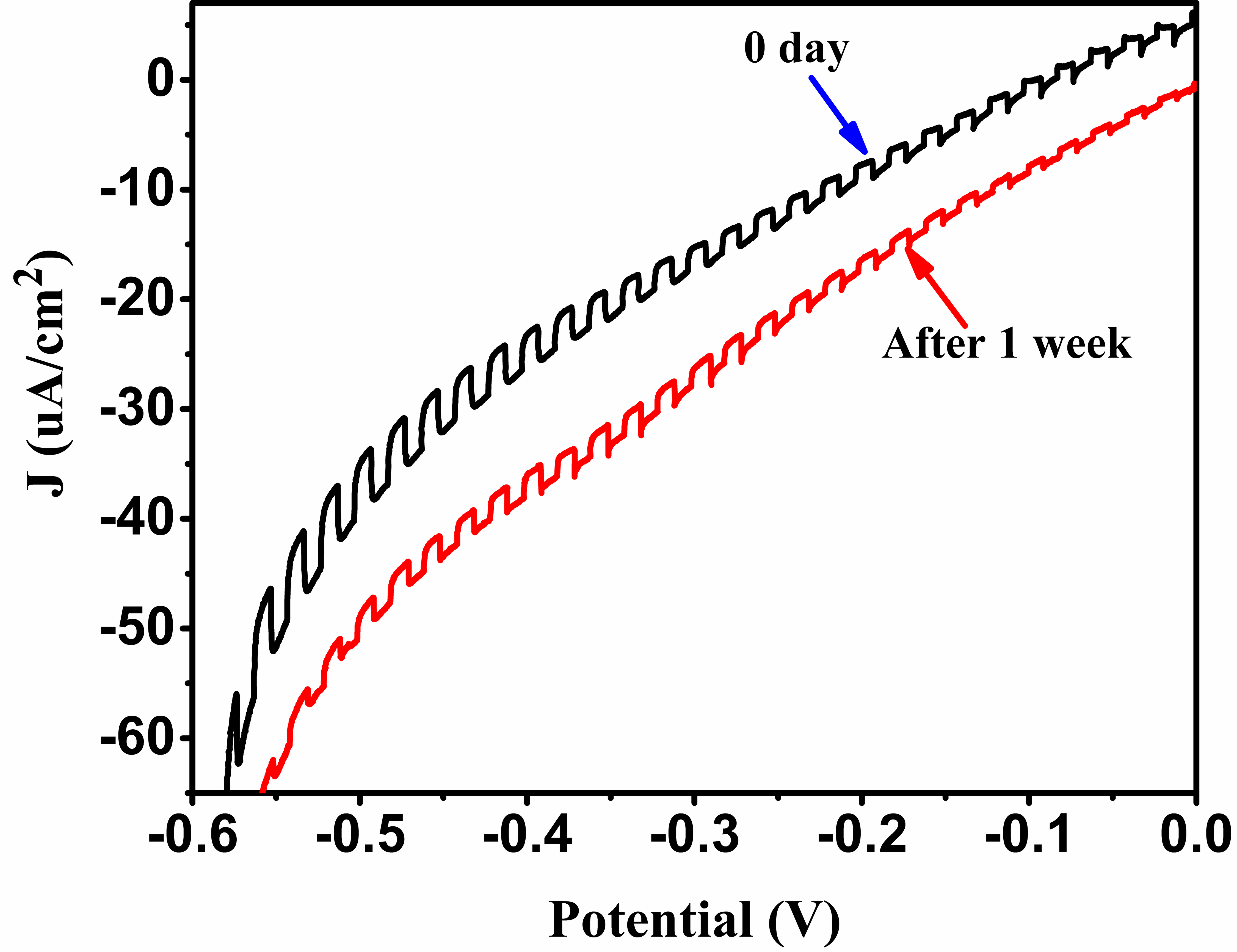

Supplement: S5 Fig — Dark line is the JV curve of fresh CVSe thin film; Red line is the JV curve of CVSe film kept in KCl aqueous solution for 1 week. (TIF) [file pone.0232184.s005.tif]

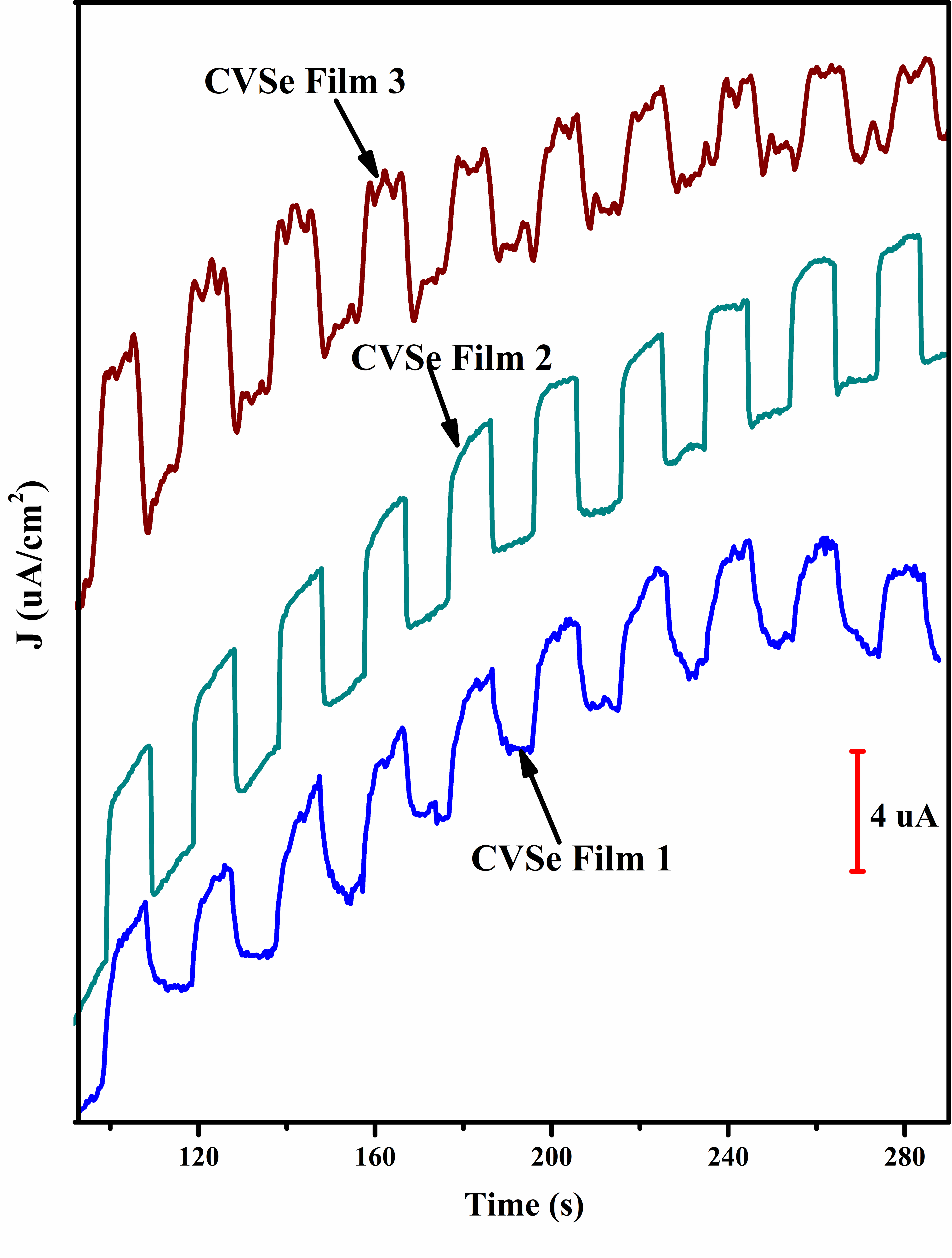

Supplement: S6 Fig — (TIF) [file pone.0232184.s006.tif]
